# Hot Deformation Behavior of Non-Alloyed Carbon Steels

**DOI:** 10.3390/ma15020595

**Published:** 2022-01-13

**Authors:** Petr Kawulok, Petr Opěla, Ivo Schindler, Rostislav Kawulok, Stanislav Rusz, Michal Sauer, Kateřina Konečná

**Affiliations:** Faculty of Materials Science and Technology, VŠB–Technical University of Ostrava, 17. Listopadu 2172/15, 70800 Ostrava, Czech Republic; petr.opela@vsb.cz (P.O.); ivo.schindler@vsb.cz (I.S.); rostislav.kawulok@vsb.cz (R.K.); stanislav.rusz2@vsb.cz (S.R.); michal.sauer@vsb.cz (M.S.); katerina.konecna@vsb.cz (K.K.)

**Keywords:** carbon steels, hot flow stress curves, dynamic recrystallization, peak flow stress, peak strain, critical strain for induce of dynamic recrystallization, activation energy at hot forming

## Abstract

The hot deformation behavior of selected non-alloyed carbon steels was investigated by isothermal continuous uniaxial compression tests. Based on the analysis of experimentally determined flow stress curves, material constants suitable for predicting peak flow stress *σ_p_*, peak strain *ε_p_* and critical strain *ε_crDRX_* necessary to induce dynamic recrystallization and the corresponding critical flow stresses *σ_crDRX_* were determined. The validity of the predicted critical strains *ε_crDRX_* was then experimentally verified. Fine dynamically recrystallized grains, which formed at the boundaries of the original austenitic grains, were detected in the microstructure of additionally deformed specimens from low-carbon investigated steels. Furthermore, equations describing with perfect accuracy a simple linear dependence of the critical strain *ε_crDRX_* on peak strain *ε_p_* were derived for all investigated steels. The determined hot deformation activation energy *Q* decreased with increasing carbon content (also with increasing carbon equivalent value) in all investigated steels. A logarithmic equation described this dependency with reasonable accuracy. Individual flow stress curves of the investigated steels were mathematically described using the Cingara and McQueen model, while the predicted flow stresses showed excellent accuracy, especially in the strains ranging from 0 to *ε_p_*.

## 1. Introduction

**Figure 1:**
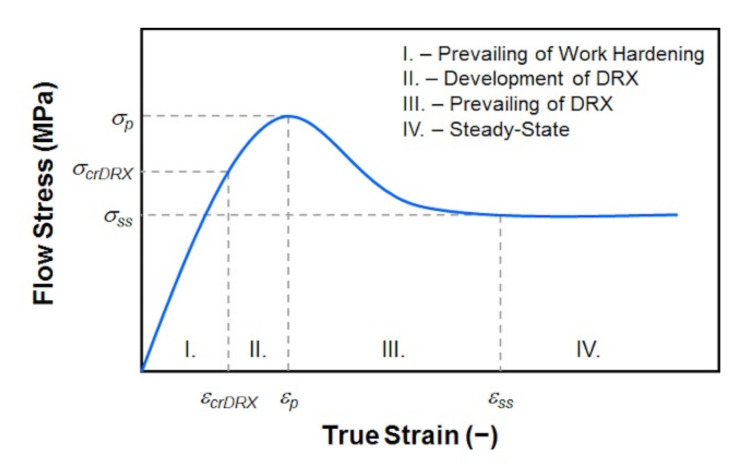
The schematic representation of flow stress curve influenced by dynamic recrystallization [12].

Dynamic recrystallization (DRX) is an important structure-forming process that occurs under the hot forming of metallic materials with low stacking fault energy [1,2,3,4,5]. This process is important, especially at the continuous hot rolling of steel strips, bars or wires [6,7,8,9,10,11]. The critical value of internal energy is required for the dynamic recrystallization initiation in the formed material, which corresponds to the achievement of critical strain *ε_crDRX_* (−) [12,13,14]. As a result, a favorable state is created for the nucleation of new grain nuclei and their growth already during the deformation of the given material. After exceeding the critical strain *ε_crDRX_*, the density of dislocations in the formed material increases, which is reflected in the flow stress increase up to a certain maximum (referred to as a peak flow stress *σ_p_* (MPa)—see Figure 1). However, a flow stress rate increment gradually decreases due to the effects of dynamic softening processes. The critical strain required to induce the dynamic recrystallization *ε_crDRX_* is thus lower than the peak strain *ε_p_*, which corresponds to the peak flow stress *σ_p_* in the flow stress–strain curve (see Figure 1). At strains higher than *ε_p_*, dynamic recrystallization prevails over the strengthening processes during the deformation, which is reflected in a decrease in flow stress until the strain corresponds to steady state *ε_ss_* (III.^rd^ region in Figure 1). After reaching the steady state, i.e., the stress *σ_ss_*, there is a balance between the softening and strengthening processes, and with increasing strain, the stress in the area IV.th does not change (see Figure 1) [12,14,15].

Depending on the chemical composition of the investigated materials and the chosen method of calculating the critical strain *ε_crDRX_*, it should apply that *ε_crDRX_* = (0.09 ÷ 0.8)·*ε_p_* [13,16,17,18]. The critical strain required to induce dynamic recrystallization increases with increasing strain rate and decreasing temperature [12,13,14,16]. The kinetics of dynamic recrystallization is also affected by the grain size of the investigated steel. The initial finer grain-sized structure significantly accelerates the occurrence of DRX because the existing fraction of grain boundaries already determine the density of nucleation sites. The number of available nucleation sites will be reduced by reducing the total area of grain boundaries in a given material volume, which will be reflected in the course of DRX [4,16,17,19,20]. Coarse-grained structure materials (e.g., in the as-cast state) show a small total grain boundary area (compared to fine-grained materials), and thus, intragranular nucleation becomes the prevailing process. Structural heterogeneities, such as deformation bands, high angle grain boundaries, and twins formed during plastic deformation also serve as sites for the formation and growth of new grain nuclei [4,17,21].

For the purposes of research of the deformation behavior of metallic materials, continuous isothermal uniaxial compression tests are very suitable, which are most often performed on the Gleeble type simulators, whose products are flow stress curves [22,23,24]. The information about maximum flow stress and kinetics of dynamic recrystallization of a given material can be obtained from these flow stress curves [2,4,16,22,25,26]. In addition, these flow stress curves can be described by mathematical models, which can then be used as one of the material’s input characteristics for a mathematical simulation using the finite element method. Several types of mathematical models have been developed for these purposes that can describe flow stress curves at high temperatures in areas of low strains (i.e., until the peak stress *σ_p_*) [27,28,29], in areas of high strains (i.e., behind the peak stress) [30,31,32] or in the whole range of applied strains (i.e., including the area of hardening and the area where dynamic recrystallization occurs) [33,34,35]. Flow stress curves can also be obtained by torsion tests, but their disadvantage is the low applicable strain rates (usually up to approx. 10 s^−1^) [36,37,38]. Higher strain rates (up to 100 s^−1^) can be achieved in the case of compression tests performed on Gleeble simulators [2,13,24,39,40]. Tensile tests are also used in some cases to determine flow stress–strain curves [41,42,43]. In these cases, however, it must be considered that during tensile deformation, a characteristic neck with difficult-to-predict geometry and complicated stress develops on the deformed specimen due to the progressive non-uniform strain. Therefore, the flow stress values determined by this method are highly inaccurate.

The main aim of the performed experiments was to study the influence of carbon content on the flow stress and kinetics of dynamic recrystallization of selected non-alloyed carbon steels. These important attainments are valuable especially from the point of view of optimization of the hot forming processes of the investigated steels, especially with respect to a continuous hot rolling. Within the presented research, the prediction of peak flow stress, peak strain, critical strain necessary to induce dynamic recrystallization (also related to peak strain), critical flow stress of the corresponding critical strain and prediction of hot deformation activation energy depending on the carbon content of non-alloyed carbon steels is solved. The secondary aim is to establish equations by which it will be possible to simply predict a critical strain for induce of dynamic recrystallization *ε_crDRX_*, directly in dependence on the peak strain *ε_p_* and also to simply predict the hot deformation activation energy only in dependence on the carbon content in the investigated steels. In addition, the approximation of measured flow stress curves of all investigated steels is also addressed.

## 2. Experiment Description

Four unalloyed carbon steels in the original as-cast state were selected for the above purposes, which differed mainly in carbon content (from 0.036 to 0.733 wt %) or carbon equivalent values (from 0.101 to 0.830)—see Table 1. In addition, steel C had a higher chromium content compared to other steels. To calculate the carbon equivalent *C_ekv_* (−), given in Table 1, the relationship proposed by the International Welding Institute was used [44]:

(1) $$C_{ekv}=C+\frac{\mathrm{Mn}}{6}+\frac{\mathrm{Cr}+\mathrm{Mo}+V}{5}+\frac{\mathrm{Cu}+\mathrm{Ni}}{15}$$

which takes into account the content of individual elements in wt %.

**Figure 2:**
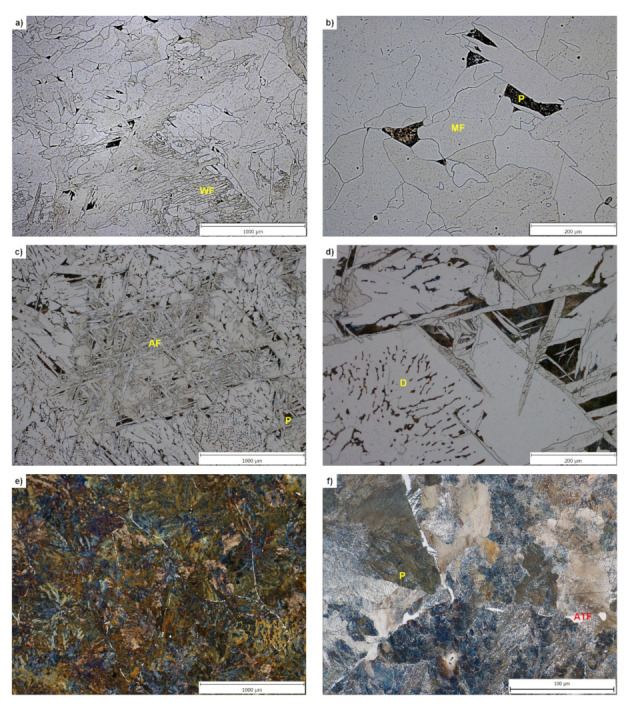
Initial structure of investigated steels A, B and D (dendrites—D, acicular ferrite—AF, allotriomorphic ferrite—ATF, massive ferrite—MF, Widmanstatten ferrite—WF, pearlite—P): (**a**,**b**) steel A; (**c**,**d**) steel B; (**e**,**f**) steel D.

The initial structural state of the investigated steels was as-cast—the steels were delivered in the form of continuously cast billets of a square cross-section of 150 × 150 mm. The initial structure of the investigated steels A, B and D (analyzed by traditional optical microscopy with use of the Olympus GX51 microscope) is shown in Figure 2. In the structure of the steel A predominates mostly massive ferrite (MF) (locally Widmanstatten ferrite (WF)) with an abnormally wide range of grain size and shape—see Figure 2a,b. The share of pearlite is very low and corresponds to the carbon content in the steel A. The structure of the steel B consists of ferrite with different morphology (including acicular ferrite (AF)) and pearlite, in some places the casting structure in the form of dendrites (D) is visible—see Figure 2c,d. The coarse pearlitic blocks in the structure of steel D are partially lined with predominantly allotriomorphic ferrite (ATF) along the boundaries of the original austenitic grains—see Figure 2e,f.

It should be noted that the steel C was not tested in this work, but for purposes of this paper, the flow stress curves obtained by isothermal compression tests carried out in the work [45] were used.

The surface layer (solidified shell) with a thickness of 10 mm was removed from the continuously cast billets. Subsequently, prisms of square cross-section (15 × 15 mm) were cut around the circumference of the continuously cast billet, from which cylindrical specimens with a diameter of 10 mm and a height of 15 mm were made, which were used for continuous isothermal compression tests.

The deformation temperatures were determined to be in the single-phase austenitic region and, at the same time, reflected the decrease of the solidus temperature and the *Ac*_3_ temperature with increasing carbon content in the investigated steels. For these reasons, with using of parametric equations [46,47], the temperature of the finish transformation ferrite to austenite *Ac*_3_ (°C) and the solidus temperature *T_S_* (°C) were first determined:

(2) $$Ac_{3}=912-370\cdot C-27.4\cdot \mathrm{Mn}+27.3\cdot \mathrm{Si}-6.35\cdot \mathrm{Cr}-32.7\cdot \mathrm{Ni}+95.2\cdot V+190\cdot \mathrm{Ti}+72.0\cdot \mathrm{Al}+64.5\cdot \mathrm{Nb}+5.57\cdot W+332\cdot S+276\cdot P+485\cdot N-900\cdot B+16.2\cdot C\cdot \mathrm{Mn}+32.3\cdot C\cdot \mathrm{Si}\;+15.4\cdot C\cdot \mathrm{Cr}+48.0\cdot C\cdot \mathrm{Ni}+4.32\cdot \mathrm{Si}\cdot \mathrm{Cr}-17.3\cdot \mathrm{Si}\cdot \mathrm{Mo}-18.6\cdot \mathrm{Si}\cdot \mathrm{Ni}+4.80\cdot \mathrm{Mn}\cdot \mathrm{Ni}+40.5\cdot \mathrm{Mo}\cdot V+174\cdot C^{2}+2.46\cdot {\mathrm{Mn}}^{2}-6.86\cdot {\mathrm{Si}}^{2}+0.322\cdot {\mathrm{Cr}}^{2}+9.90\cdot {\mathrm{Mo}}^{2}+1.24\cdot {\mathrm{Ni}}^{2}-60.2\cdot V^{2}$$

(3) $$T_{S}=1535-200\cdot C-183.9\cdot S-124.5\cdot P\;-6.8\cdot \mathrm{Mn}-12.3\cdot \mathrm{Si}-4.1\cdot \mathrm{Al}-1.4\cdot \mathrm{Cr}-4.3\cdot \mathrm{Ni}$$

The determined temperatures *Ac*_3_ and *T_S_* of the steels A, B and D are given in Table 2. The lowest deformation temperature was chosen so that about 40–50 °C was added to the calculated temperature *Ac*_3_ (see Table 2). The highest deformation temperature was determined by subtracting about 270–300 °C from the *T_S_* solidus temperature (see Table 2). The remaining deformation temperatures *T_d_* (°C), were chosen with a temperature step of 100 °C. The highest deformation temperature then represented the temperature of uniform austenitization of the specimens for the given investigated steel.

Uniaxial continuous isothermal compression tests were performed on a Gleeble 3800-GTC universal hot deformation simulator using a Hydrawedge II unit (Dynamic Systems Inc., Poestenkill, NY, USA [48]).

The prepared cylindrical specimens were uniformly electrically resistance preheated (at a heating rate of 10 °C·s^−1^) to the selected austenitization temperatures (steel A—1250 °C, steel B—1200 °C, steel D—1100 °C). After 60 s holding time they were cooled at a rate of 5 °C·s^−1^ to selected deformation temperatures (see Table 2), after which a 30 s holding time was included due to homogenization of the temperature in the specimen volume. Subsequently, the specimens were deformed by uniaxial compression to a true strain of 1.0 at constant strain rates of 0.05 s^−1^, 1 s^−1^ and 20 s^−1^.

Steel C in the initial as-cast state was deformed in work [45] by uniaxial compression on the same equipment at the same deformation parameters (true strain of 1.0 at constant strain rates 0.05 s^−1^, 1 s^−1^ and 20 s^−1^), but deformation temperatures were chosen differently. The steel C specimens were uniformly preheated to 1250 °C and after a subsequent 60 s holding were cooled at a rate of 5 °C·s^−1^ to the selected deformation temperatures (900 °C, 1000 °C, 1120 °C, 1250 °C), after which it was reached before deformation also 30 s holding time [45].

The temperature of the specimens was measured during the compression tests with one pair of K-type thermocouple wires, which were surface welded to the center of the height of the tested specimen. All compression tests were performed in a vacuum to eliminate the oxidation of specimens.

## 3. Calculation Methods

The Zener–Hollomon parameter *Z* (s^−1^), which is defined as the temperature-compensated strain rate, can be used to predict *σ_p_* and *ε_p_* [4,24,26,49]:

(4) $$Z=\dot{\varepsilon }\cdot \exp\left(\frac{Q}{R\cdot T}\right)$$

(5) $$\sigma_{p}=\frac{1}{\alpha }\cdot \mathrm{arcsinh}\sqrt[{n}]{\frac{Z}{A}}$$

(6) $$\varepsilon_{p}=U\cdot Z^{W}$$

where $\dot{\varepsilon }$ (s^−1^) is the strain rate, *Q* (J∙mol^−1^) is the hot deformation activation energy, *R* is the molar gas constant (8.314 J·mol^−1^·K^−1^), *T* (K) is the deformation temperature, *α* (MPa^−1^), *A* (s^−1^), *n* (−), *U* (s) a *W* (−) are the material constants.

The activation energy brings the material factor into relation (4), i.e., the influence of chemical composition, structure, etc. The value of the activation energy *Q*, together with other material constants *α* (MPa^−1^), *A* (s^−1^), *n* (−) suitable for prediction of the peak flow stress according to Equation (5), can be determined by regression analysis of the known Garofalo sinus-hyperbolic relation [50]:

(7) $$\dot{\varepsilon }=A\cdot \exp\left(-\frac{Q}{R\cdot T}\right)\cdot {\left[\sin h\left(\alpha \cdot \sigma_{p}\right)\right]}^{n}$$

where *σ_p_* (MPa) is peak flow stress, which corresponds to *ε_p_*. Equation (7) is often solved by a simple graphical method based on repeatedly used linear regression [24,26,51]. For high deformation temperatures or low values of flow stress, relation (7) can be adjusted to the power function:

(8) $$\dot{\varepsilon }=A_{1}\cdot \exp\left(-\frac{Q}{R\cdot T}\right)\cdot \sigma_{p}{}^{n}$$

For low deformation temperatures or high values of flow stress, relation (7) can be transformed into the exponential form:

(9) $$\dot{\varepsilon }=A_{2}\cdot \exp\left(-\frac{Q}{R\cdot T}\right)\cdot \exp\left(\beta \cdot \sigma_{p}\right)$$

where *A*_1_, *A*_2_ a *β* are the material constants. The following relation gives the constant α from Equation (7):

(10) $$\alpha =\frac{\beta }{n}$$

To determine the values of material constants *n*, *α*, *A*, *Q*, *U* and *W* of all investigated steels, the verified software ENERGY 4.0 was used. The software ENERGY 4.0 works on the basis of a combination of partial linear regressions and final complex nonlinear regression, thanks to which these constants determined in the previous step by linear regressions, are further specified [4,24,25,26,52]. In several works, only the simplified power Equation (8) was used to determine the activation energy and subsequently to predict peak flow stress *σ_p_*, and the calculation was, therefore, significantly simpler—see, e.g., [53,54,55]. However, this simplification has been shown to lead to inaccurate results [24]. The calculation of hot deformation activation energy from experimental values by solving the sinushyperbolic Equation (7) is a proven method that has been successfully applied to several types of materials, e.g., steels [4,5,24,37,40], alloys based on copper [25], aluminum [26,56], magnesium [24,52] or nickel [2,57,58].

The critical strain *ε_crDRX_* (−) necessary to induce dynamic recrystallization can be determined metallographically; however, this approach is extremely long, and the results are inaccurate. It is more appropriate to use a mathematical approach based on the analysis of the dependence of the work hardening rate on the stress in the range of strains from zero to peak. The work hardening rate *θ* (MPa) is defined as stress derivation according to deformation [18]:

(11) $$\theta =\frac{d\sigma }{d\varepsilon }$$

The inflexion point of the curve *θ*~*σ* corresponds to the onset of DRX. The global minimum on the curve expressing the dependence *dθ*/*dσ*~*σ* corresponds to the inflexion point of the curve *θ*~*σ* and thus corresponds to the beginning of DRX [13,18,59,60]. From a mathematical point of view, this global minimum (on the curve *dθ*/*dσ*~*σ*) is the zero value of the second derivative *θ* according to *σ* [18]:

(12) $$\frac{\partial^{2}\theta_{c}}{\partial \sigma^{2}}=0$$

where *θ_c_* is the work hardening rate in the critical state for the onset of DRX. The inflexion point of the curve *θ*~*σ* is the same as the inflexion point on the curve expressing the dependence ln *θ*~*ε* and, therefore, must apply [18]:

(13) $${\left.\frac{\partial^{2}\theta_{c}}{\partial \sigma^{2}}\right|}_{\sigma =\sigma_{crDRX}}={\left.\frac{\partial^{2}\ln\theta_{c}}{\partial \varepsilon^{2}}\right|}_{\varepsilon =\varepsilon_{crDRX}}=0$$

To derive an equation suitable for the prediction of critical strain *ε_crDRX_*, a model by Cingara and McQueen was chosen in this case, which allows a mathematical description of flow stress curves up to the peak point (up to the strain of *ε_p_*) [27]:

(14) $$\sigma =\sigma_{p}\cdot {\left[\frac{\varepsilon }{\varepsilon_{p}}\cdot \exp\left(1-\frac{\varepsilon }{\varepsilon_{p}}\right)\right]}^{C}$$

where *ε* (−) is a value of true strain, and *C* (−) is the strain hardening exponent. The logarithm of both sides of Equation (14) leads to the following linear equation [18]

(15) $$\ln\left(\frac{\sigma }{\sigma_{p}}\right)=C\cdot \left[\ln\left(\frac{\varepsilon }{\varepsilon_{p}}\right)+\left(1-\frac{\varepsilon }{\varepsilon_{p}}\right)\right]$$

Linear regression can then be used to determine the required values of the hardening exponent *C* separately from Equation (15) for each combination of temperature and strain rate (for one flow stress curve). For practical use, it is still necessary to compile a mathematical description of the exponent *C*, which will be described later. By deriving relation (14) according to the strain *ε*, the expression for determining *θ* can be obtained. By substituting the expression *θ* into Equation (13) and its subsequent solution, it is possible to arrive at a final expression, which allows the determination of the critical strain *ε_crDRX_* [13,18]:

(16) $$\varepsilon_{crDRX}=\varepsilon_{p}\cdot \frac{\sqrt{1-C}+C-1}{C}$$

Therefore, Equation (16), intended for the prediction *ε_crDRX_*, depends on the peak strain *ε_p_* and on the strain hardening exponent *C*.

## 4. Analysis of Measured Data

### 4.1. Prediction of Peak Flow Stress and Peak Strain

**Figure 3:**
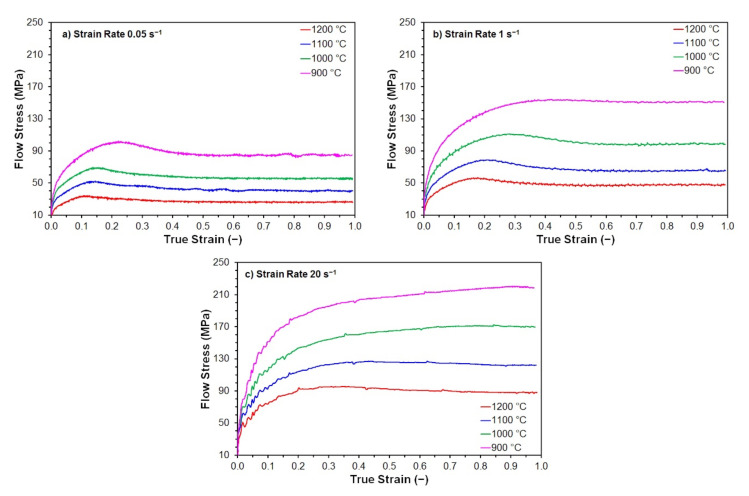
The measured flow stress curves of steel B: (**a**) strain rate 0.05 s^−1^; (**b**) strain rate 1 s^−1^; (**c**) strain rate 20 s^−1^.

Flow stress curves were obtained by continuous isothermal uniaxial compression tests (see example in Figure 3). The coordinates of stress peaks were determined from these flow stress curves, which were then used as input data to calculate activation energy and other material constants suitable for predicting peak flow stress *σ_p_* and peak strain *ε_p_* during hot forming of the investigated steels.

**Figure 4:**
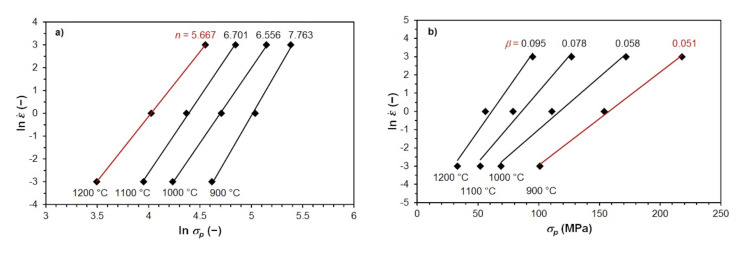

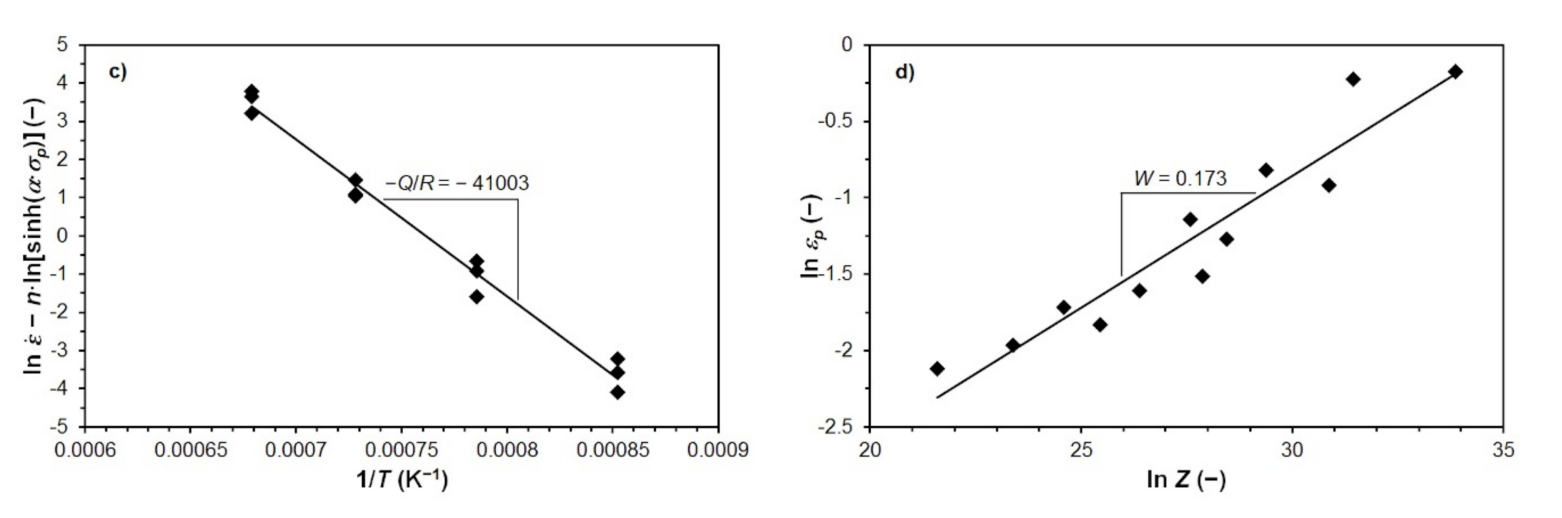
Calculation of material constants in Equations (7) and (6) for steel B: (**a**) calculation of *n* = 5.667; (**b**) calculation of *β* = 0.051; (**c**) calculation of *Q* = 341 kJ∙mol^−1^; (**d**) determination of *W* = 0.173.

The constant *n* (see Equation (8)) for the selected high-temperature level is determined by the linear regression of the experimentally determined values of *σ_p_* in the coordinates ln $\dot{\varepsilon }$~ln *σ_p_* (see Figure 4a). For the selected low-temperature level, the constant *β* is determined by linear regression in the coordinates ln $\dot{\varepsilon }$~*σ_p_* (see Equation (9) and Figure 4b). After calculating the material constant *α* according to Equation (10), the final linear regression of all measured data plotted in the coordinates ln $\dot{\varepsilon }$ − *n* ln [sinh (*α*⋅*σ_p_*)]~*T*, material constants *Q* and *A* can be determined (see Figure 4c). The determined material constants are then refined by the final gradient optimization algorithm by solving Equation (7) via nonlinear regression analysis, which includes two mutually independent variables (temperature and strain rate). The material constants *U* and *W* are then determined using the activation energy by linear regression of the experimentally determined *ε_p_* values in the coordinates ln *ε_p_*~ln *Z* (see Figure 4d). The values of material constants of the examined steels, determined by regression analysis in the ENERGY 4.0 program, are given in Table 3.

The determined material constants *Q*, *n*, *α*, *A*, *U* and *W* (see Table 3) can be used for the given thermomechanical forming parameters for the prediction of peak flow stress *σ_p_* and peak strain *ε_p_* of investigated steels according to Equations (5) and (6). The accuracy of the predicted values *σ_p_* and *ε_p_* was simply evaluated using the correlation coefficient *R* (−), the relative calculation error Δ (%) and its mean values Δ_mean_ (%)—see Table 4 and Table 5. The relative error values were determined according to the following relationships:

(17) $$\Delta =\frac{\left(\sigma_{p\left(measured\right)}-\sigma_{p\left(predicted\right)}\right)}{\sigma_{p\left(measured\right)}}\cdot 100$$

(18) $$\Delta =\frac{\left(\varepsilon_{p\left(measured\right)}-\varepsilon_{p\left(predicted\right)}\right)}{\varepsilon_{p(\left(measured\right)}}\cdot 100$$

**Figure 5:**
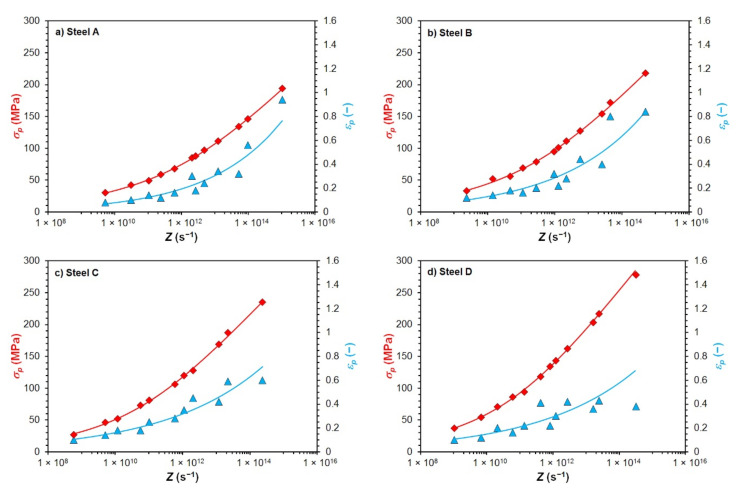
Comparison of measured and predicted values of *σ_p_* and *ε* depending on the parameter *Z* (points—measured values; lines—predicted values): (**a**) steel A; (**b**) steel B; (**c**) steel C; (**d**) steel D.

The excellent agreement of the calculated and measured values of the peak flow stress *σ_p_* is confirmed by high values of correlation coefficients *R* and very low values of mean calculation error Δ_mean_ given in Table 4. In the case of the peak strain *ε_p_* the accuracy of back-calculation was lower but entirely sufficient—see Table 5. A comparison of experimentally determined and according to Equations (5) and (6) predicted values of *σ_p_* a *ε_p_* depending on the Zener–Hollomon parameter of all investigated steels is shown in Figure 5. The results shown in Figure 5, Table 4 and Table 5 thus confirm that the chosen procedure for predicting the values of *σ_p_* and *ε_p_* was correct, respectively that Equations (5) and (6) (together with the constants shown in Table 3) can be used to predict the *σ_p_* and *ε_p_* of all investigated steels at given thermomechanical conditions of forming.

### 4.2. Prediction of Critical Flow Stress and Critical Strain for Induce of DRX

**Figure 6:**
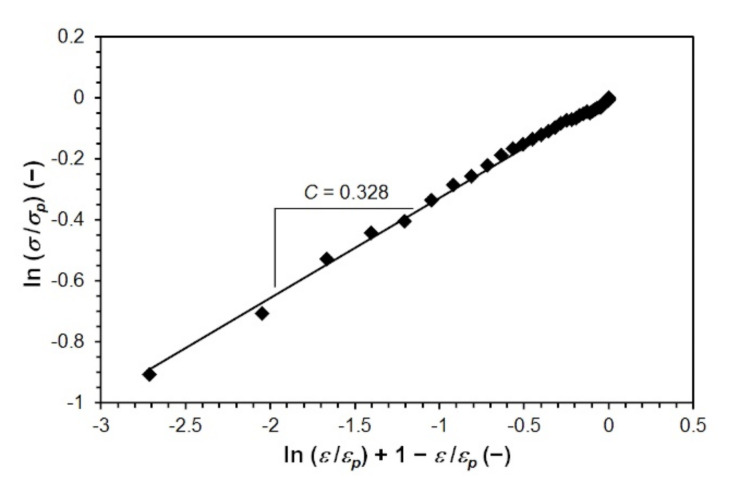
Determining the constant *C* for steel B (temperature 1000 °C, strain rate 20 s^−1^).

To predict the critical strain *ε_crDRX_* (−) necessary to induce dynamic recrystallization, it is necessary to derive a relationship that will allow the prediction of strain hardening exponent *C* (see Equation (16)). The value of the strain hardening exponent *C* is determined for each measured flow stress curve (i.e., for each combination of temperature and strain rate) as the line slope (dependence (15)), considering the zero value of the intersection with the vertical axis. An example of determining the constant *C* by linear regression of the measured data for one combination of temperature and strain rate (1000 °C and 20 s^−1^) for steel B is shown in Figure 6.

**Figure 7:**
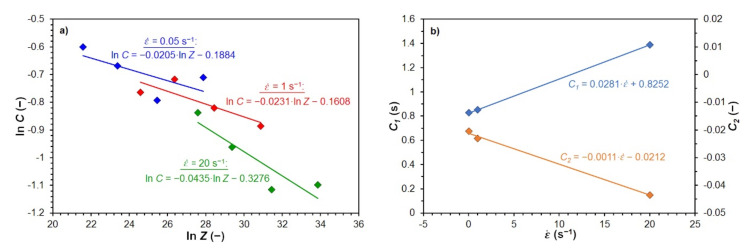
Determination of constants *C*_1_ and *C*_2_ for steel B: (**a**) determination of parameters *C*_1_ and *C*_2_ according to the Equation (19); (**b**) dependence of parameters *C*_1_ and *C*_2_ on strain rate.

The prediction of the strain hardening exponent *C* is possible based on its functional dependence on the Zener–Hollomon parameter *Z* (4), which includes the interaction effects of strain rate and temperature. The dependence of exponent *C* on parameter *Z* has a power character for a constant value of a strain rate and a variable deformation temperature [13]:

(19) $$C=C_{1}\cdot Z^{C_{2}}$$

where *C_1_* (s) and *C_2_* (−) are the material parameters, which are determined by regression separately for each strain rate $\dot{\varepsilon }$ (as line slope and the intersection of the given dependence with the *y*-axis), by converting relation (19) into a linear form—see Figure 7a (for steel B). The parameters obtained in this way are further linearly dependent on the strain rate $\dot{\varepsilon }$ [30]—see Figure 7b (for steel B). The resulting relationship for the prediction of the strain hardening exponent *C* will, therefore, have the general form:

(20) $$C=\left(a\cdot \dot{\varepsilon }+b\right)\cdot Z^{\left(c\cdot \dot{\varepsilon }+d\right)}$$

The values of material constants *a* (s^2^), *b* (−), *c* (s) and *d* (−) intended for the prediction of the strain hardening exponent *C* of all investigated steels (according to relation (20)) are given in Table 6.

Using Equations (4), (6), (16) and (20) and using the material constants listed in Table 3 and Table 6. It can be predicted the critical *ε_crDRX_* strains necessary to induce dynamic recrystallization of all investigated non-alloyed carbon steels for the given thermomechanical forming conditions. The critical flow stress *σ_crDRX_* (MPa), corresponding to the onset of dynamic recrystallization, can then be relatively easily determined using a modified Cingara and McQueen relationship (14), in which the strain *ε* is replaced by the critical strain required to induce dynamic recrystallization *ε_crDRX_*:

(21) $$\sigma_{crDRX}=\sigma_{p}\cdot {\left[\frac{\varepsilon_{crDRX}}{\varepsilon_{p}}\cdot \exp\left(1-\frac{\varepsilon_{crDRX}}{\varepsilon_{p}}\right)\right]}^{C}$$

**Figure 8:**
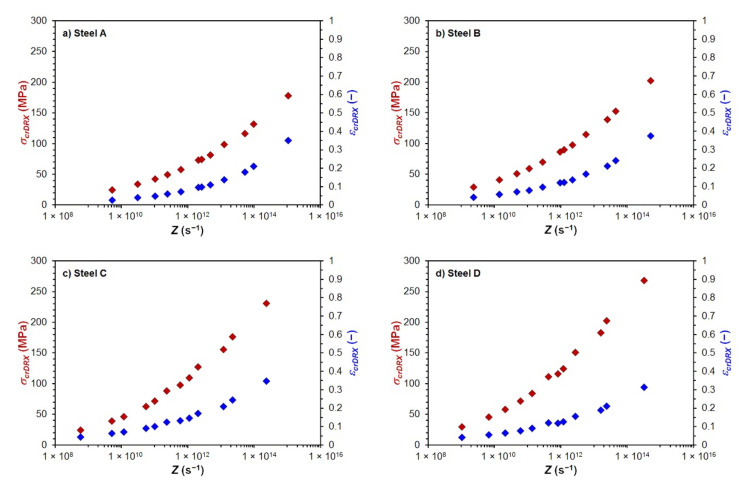
Dependence of determined values *σ_crDRX_* and *ε_crDRX_* on parameter *Z*: (**a**) steel A; (**b**) steel B; (**c**) steel C; (**d**) steel D.

The course of the determined values *σ_crDRX_* and *ε_crDRX_* depending on the Zener–Hollomon parameter of all investigated steels is shown in Figure 8.

## 5. Verification of Determined Critical Strains *ε_crDRX_*

To verify the validity of the determined critical strains necessary to induce dynamic recrystallization *ε_crDRX_*, additional isothermal uniaxial compression tests were performed on steels A, B and D at selected temperature and strain rate combinations. These additional experiments are aimed to detect dynamically recrystallized grains in the microstructure of the investigated steels. The heating mode of individual steels, or cylindrical specimens with a diameter of 10 mm and a height of 15 mm, corresponded to the description given in Section 2. After heating and defined holding time at the deformation temperature, the specimens were deformed by uniaxial compression. The strain value was determined to be a multiple of 1.3·*ε_crDRX_*, while the critical strain necessary to induce dynamic recrystallization of *ε_crDRX_* was determined according to relation (16), resp. according to the procedure described in Section 4.2. In order to preserve the structure, the specimens were quenched in water immediately after deformation. The thermomechanical parameters of the individual supplementary uniaxial compression tests are given in Table 7.

**Figure 9:**
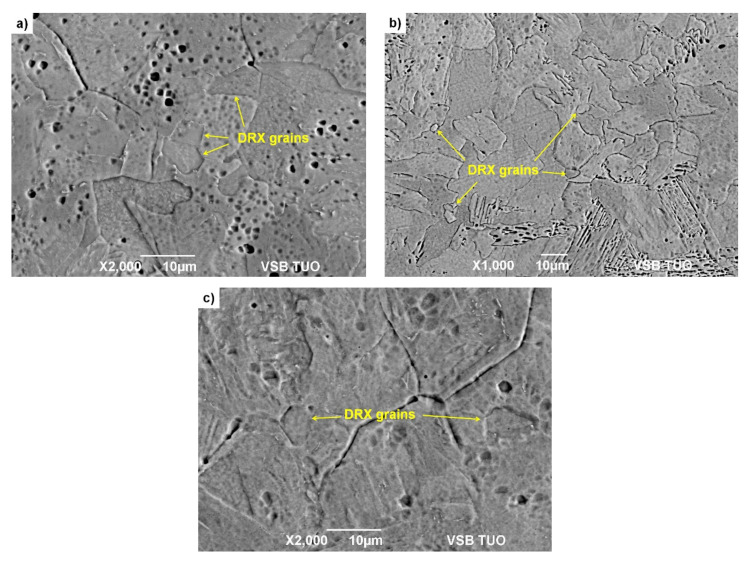
Metallographic images with etched boundaries of original austenitic grains of steel A: (**a**) *T_d_* = 950 °C, $\dot{\varepsilon }$ = 0.05 s^−1^, *ε* = 0.13; (**b**) *T_d_* = 1050 °C, $\dot{\varepsilon }$ = 1 s^−1^, *ε* = 0.14; (**c**) *T_d_* = 1150 °C, $\dot{\varepsilon }$ = 20 s^−1^, *ε* = 0.18.

**Figure 10:**
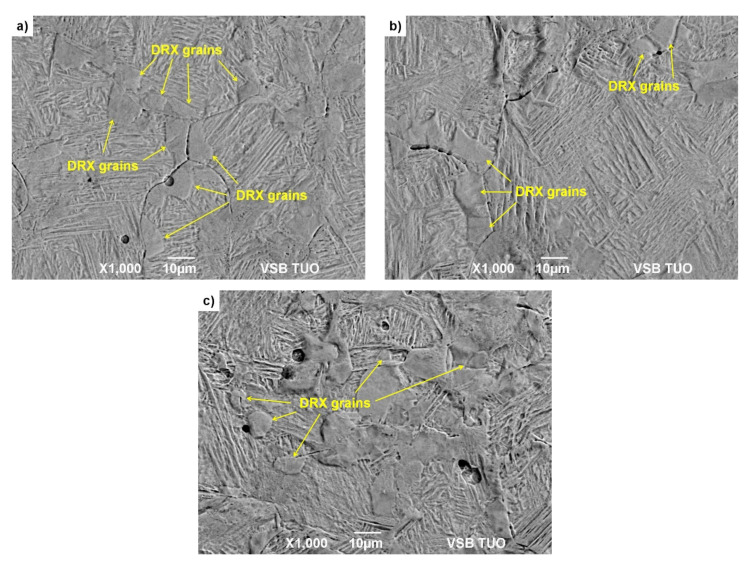
Metallographic images with etched boundaries of original austenitic grains of steel B: (**a**) *T_d_* = 900 °C, $\dot{\varepsilon }$ = 0.05 s^−1^, *ε* = 0.16; (**b**) *T_d_* = 1000 °C, $\dot{\varepsilon }$ = 1 s^−1^, *ε* = 0.18; (**c**) *T_d_* = 1100 °C, $\dot{\varepsilon }$ = 20 s^−1^, *ε* = 0.22.

The deformed and quenched specimens were then tempered at 300 °C. The specimens were cut in the middle of their diameter (in the direction of their height) into two halves for metallographic analysis. The specimens prepared in this way were then etched with Alkilo with the addition of HCl to highlight the boundaries of the original austenitic grains. The microstructure in the middle of the height of these specimens was documented by SEM analysis on a JEOL JSM-6490LV microscope under BES imaging (combination of backscattered and secondary electrons, i.e., material and topographic contrast). Documentation of the microstructure of the examined specimens from steels A and B is given in Figure 9 and Figure 10.

Using SEM analysis of uniaxial compression tested specimens, fine dynamically recrystallized grains were detected in the microstructure of the steels A and B in all investigated cases—see Figure 9 and Figure 10. These dynamically recrystallized grains, in most cases, are formed at the boundaries of the original austenitic grains (see for example, Figure 9b, Figure 10a or Figure 10b). In the case of the steel B, the differences between the original and dynamically recrystallized grains are clearly visible—see Figure 10. Due to the quenching of the deformed specimens with water, martensite with relatively coarse lath was formed in the original austenitic grains. The new dynamically recrystallized grains, formed at the boundaries of the original austenitic grains, contained martensite with a finer morphology. This additional experiment thus confirmed the validity of the determined critical strains *ε_crDRX_*, which are necessary to induce dynamic recrystallization of steels A and B.

**Figure 11:**
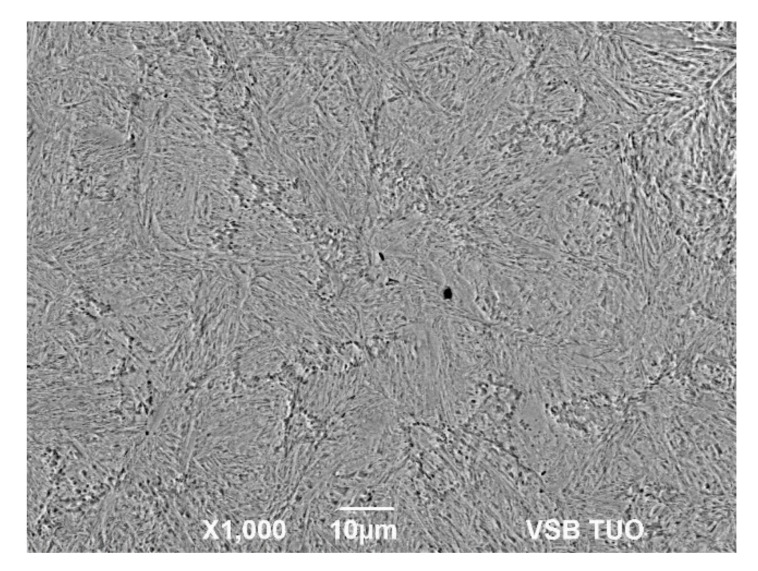
Metallographic image with uncertain etched boundaries of the original austenitic grains of steel D—*T_d_* = 1100 °C, $\dot{\varepsilon }$ = 20 s^−1^, *ε* = 0.16.

Unfortunately, in the case of the steel D, the boundaries of the original austenitic grains could not be clearly etched, not even with the use of other etchants. Signs of the boundaries of the original austenitic grains could only be seen in specimen D3 (see Figure 11), but it is not possible to reliably determine whether these are dynamically recrystallized grains.

## 6. Discussion of Results

### 6.1. Predicted Peak Flow Stress and Peak Strain

**Figure 12:**
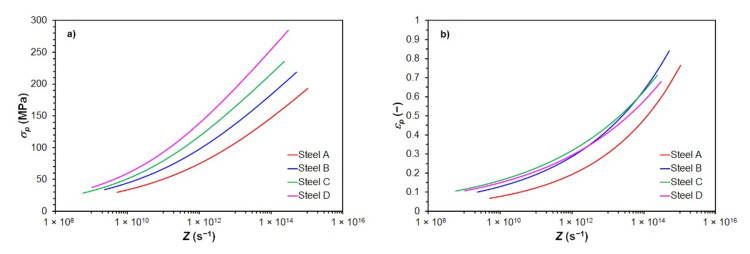
The effect of Zener–Hollomon parameter *Z* to the predicted values of *σ_p_* and of *ε_p_* of investigated steels: (**a**) peak flow stress *σ_p_*; (**b**) peak strain *ε_p_*.

Using Equations (4) and (6) and using the material constants listed in Table 3, peak flow stress *σ_p_* and peak strain *ε_p_* of the investigated steels for the given thermomechanical forming conditions can be predicted—see Figure 12. The peak flow stress *σ_p_* and the peak strain *ε_p_* of the investigated steels increases with the increasing value of the Zener–Hollomon parameter—see Figure 12. Steels with a higher carbon content or a higher carbon equivalent value showed greater maximum flow stress for a given size of the Zener–Hollomon parameter—see Figure 12a. In the case of the influence of carbon content or carbon equivalent on the value of the peak strain *ε_p_*, the situation is more complicated. The low carbon steel A showed the lowest peak strain *ε_p_* of all investigated steels—see Figure 12b. Thus, it can be assumed that for these conditions, the lowest strain will be needed to induce dynamic recrystallization of this low carbon steel compared to other investigated steels. In the case of the medium carbon steel C, for high values of the parameter *Z* (above 1 × 10^14^ s^−1^), smaller strains are needed to achieve peak flow stress than in the case of the low carbon steel B—see Figure 12b. In the case of the high carbon steel D, the values of predicted peak strain *ε_p_* in the whole range of the parameter *Z* are slightly lower than in medium carbon steel C. At values of the *Z* parameter above 4 × 10^12^ s^−1^, the predicted peak strain *ε_p_* of the steel D is also lower than in the low carbon steel B—see Figure 12b.

### 6.2. Predicted Critical Flow Stress and Critical Strain for Induce of DRX

**Figure 13:**
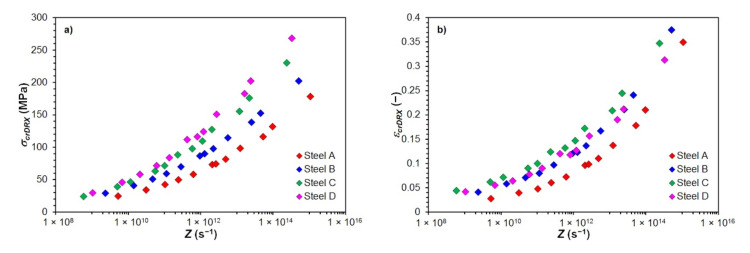
The effect of Zener–Hollomon parameter *Z* to determined critical stresses *σ_crDRX_* and critical strains *ε_crDRX_* for induce of dynamic recrystallization of investigated steels: (**a**) critical flow stress *σ_crDRX_*; (**b**) critical strain *ε_crDRX_*.

The comparison of the determined critical flow stresses *σ_crDRX_* and critical strains *ε_crDRX_* necessary to induce dynamic recrystallization, depending on the Zener–Hollomon parameter *Z*, is shown for all investigated steels in Figure 13. Steels with higher carbon content or a higher value of carbon equivalent for a given size of the Zener–Hollomon parameter, showed larger values of critical flow stress *σ_crDRX_*—see Figure 13a, similar to the case of peak flow stress *σ_p_*. Influence of carbon content or carbon equivalent to the value of the critical strain *ε_crDRX_* was, except for the high carbon steel D, similar to the critical flow stress *σ_crDRX_*. For a similar size of the Zener–Hollomon parameter, smaller critical strains *ε_crDRX_* are required to induce dynamic recrystallization of the high carbon steel D than in the case of the medium carbon steel C—see Figure 13b.

Figure 12b and Figure 13b document that in the case of the steels B, C and D, in comparison to the steel A, larger strains are needed to induce the dynamic recrystallization. This would suggest that a higher carbon content in the investigated steels should lead to a retardation of dynamic softening processes. However, when comparing the B, C and D steels only among themselves, the effect of the carbon content on the kinetics of dynamic recrystallization is not convincingly clear because the corresponding values of the critical strain *ε_crDRX_* as well as the values of the peak strain *ε_p_* are very similar (for given values of the parameter *Z*).

The graphical dependencies shown in Figure 12 and Figure 13 confirm that the stress *σ_p_* or *σ_crDRX_* and the strain *ε_p_* or *ε_crDRX_* increase in all investigated steels, with the increasing value of the parameter *Z*. This confirms the well-known fact that it is necessary to apply larger strains to induce dynamic recrystallization of the material at high values of the parameter *Z* [2,4,5,40,57], or it is necessary to ensure their accumulation during very short inter-pass times (for example, during rolling strips or in finishing blocks in wire rolling) [7,10].

**Figure 14:**
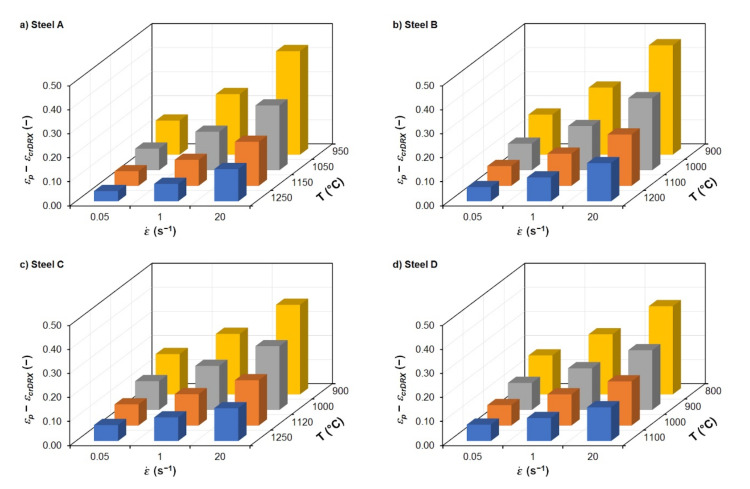
Graphical comparison of the difference between the peak strain *ε_p_* and the critical strain *ε_crDRX_* of the investigated steels: (**a**) steel A; (**b**) steel B; (**c**) steel C; (**d**) steel D.

Figure 14 shows 3D column charts documenting the dependence of the difference between peak strain *ε_p_* and critical strain *ε_crDRX_* for the given thermomechanical forming conditions of all investigated steels. It is clear from Figure 14 that the difference between *ε_p_* and *ε_crDRX_* increases as the deformation temperature decreases and the strain rate increases. At high deformation temperatures and low strain rates, the difference between *ε_p_* and *ε_crDRX_* is small (approx. 0.04 to 0.07). However, in the case of a combination of low deformation temperatures and high strain rates, the difference between *ε_p_* and *ε_crDRX_* reaches values up to 0.46, which is a significant fact, especially from the point of view of operating conditions. However, the ratio between *ε_crDRX_* and *ε_p_* is very similar for all combinations of thermomechanical forming conditions of all investigated steels—i.e., ranging from 38% to 48%.

**Figure 15:**
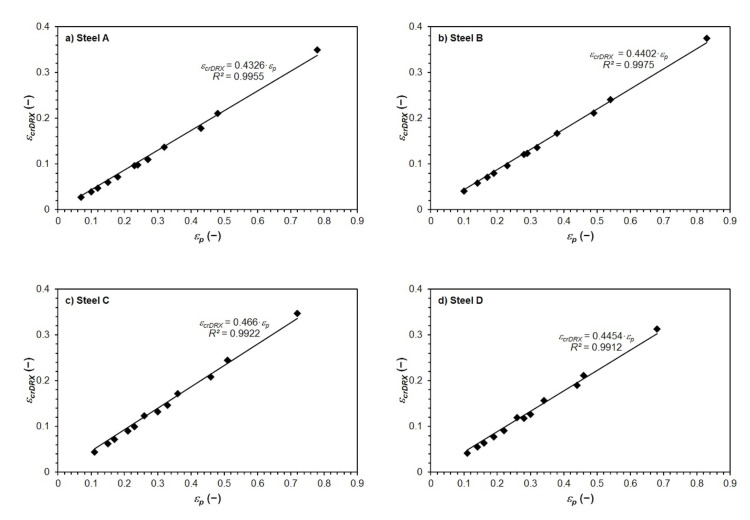
Relationship between critical strain *ε_crDRX_* and peak strain*ε_p_* of investigated steels: (**a**) steel A; (**b**) steel B; (**c**) steel C; (**d**) steel D.

The procedure for determining the critical strains *ε_crDRX_* required to induce dynamic recrystallization, which is given in Section 4.2, is relatively time-consuming. However, the determined critical strains *ε_crDRX_* can be related to the predicted peak strain *ε_p_*—see Figure 15. It is, therefore, clear from the Figure 15 that the predicted critical strain *ε_crDRX_* depends linearly on the peak strain *ε_p_*. Thus, for the investigated non-alloyed carbon steels, it is possible, very simply and at the same time with very good accuracy, to determine the value of the critical strain *ε_crDRX_* only via relation to the value of the peak strain *ε_p_*:

(22) $$\mathrm{for}\;\mathrm{steel}\;A:\;\varepsilon_{crDRX}=0.4326\cdot \varepsilon_{p}$$

(23) $$\mathrm{for}\;\mathrm{steel}\;B:\;\varepsilon_{crDRX}=0.4402\cdot \varepsilon_{p}$$

(24) $$\mathrm{for}\;\mathrm{steel}\;C:\;\varepsilon_{crDRX}=0.466\cdot \varepsilon_{p}$$

(25) $$\mathrm{for}\;\mathrm{steel}\;D:\varepsilon_{crDRX}=0.4454\cdot \varepsilon_{p}$$

The very good accuracy of the above listed equations is documented by the high values of the coefficients of determination: for Equation (22) *R*^2^ = 0.9955; for Equation (23) *R*^2^ = 0.9975; for Equation (24) *R*^2^ = 0.9922 and for Equation (25) *R*^2^ = 0.9912.

**Figure 16:**
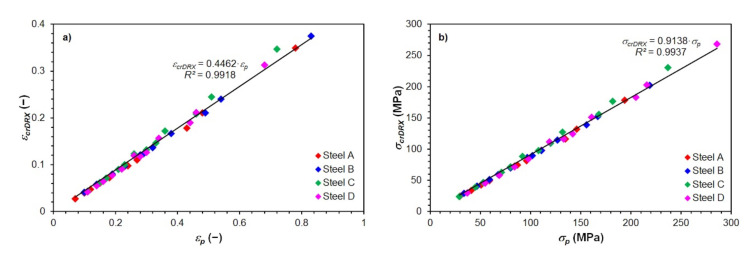
Dependence of the critical strain *ε_crDRX_* and the critical flow stress *σ_crDRX_* on the corresponding coordinates of the peak stress of all investigated steels: (**a**) critical strain *ε_crDRX_*; (**b**) critical flow stress *σ_crDRX_*.

Since, in Equations (22)–(25), the intersection with the *y*-axis is equal to 0, and the slope of the above linear dependencies of the critical strain *ε_crDRX_* on the peak strain *ε_p_* of all investigated steels are very similar (see Equations (22)–(25)), it is possible to compile a relationship that would describe this dependence (see Figure 16a) comprehensively for all investigated steels:

(26) $$\varepsilon_{crDRX}=0.4462\cdot \varepsilon_{p}$$

Similarly, it is also possible to approach the simplified prediction of critical flow stress *σ_crDRX_* in dependence on the peak flow stress *σ_p_* for all investigated steels using a single complex equation (see Figure 16b):

(27) $$\sigma_{crDRX}=0.9138\cdot \sigma_{p}$$

The excellent accuracy of Equation (26) is documented by the high value of the corresponding coefficient of determination *R*^2^ = 0.9918 and graphically in Figure 16a. In the case of Equation (27), its excellent accuracy is again documented by the high value of the corresponding coefficient of determination *R*^2^ = 0.9937, as well as graphically in Figure 16b.

Linear dependence of the critical strain *ε_crDRX_* on the peak strain *ε_p_* of all investigated steels is expressed with excellent accuracy by Equations (22)–(26). Equation (24), which expresses the linear dependence of *ε_crDRX_* on *ε_p_* of the medium carbon steel C, corresponds very well to the analogous dependence derived in [13] for C45 medium carbon steel (with similar chemical composition), which, however, was deformed in a wider range of deformation temperatures and strain rates:

(28) $$\varepsilon_{crDRX}=0.48\cdot \varepsilon_{p}$$

The relationship expressing the dependence of critical strain *ε_crDRX_* on peak strain *ε_p_* was also derived by Liu et al. [18] for 316LN high-alloy steel:

(29) $$\varepsilon_{crDRX}=0.6\cdot \varepsilon_{p}$$

The 316LN steel used in [18] contained 0.12 C, 1.28 Mn, 13.2 Ni, 17.2 Cr and 2.4 Mo (all in wt %). Fernández et al. in [17] also derived a relation describing the linear dependence of critical strain *ε_crDRX_* on peak strain *ε_p_* for low carbon micro-alloyed steels:

(30) $$\varepsilon_{crDRX}=0.77\cdot \varepsilon_{p}$$

The micro-alloyed steels used in [17] contained 0.1 C, 1.28 Mn, 0.035 Nb, 0.0053 N and 0.07 C, 0.62 Mn, 0.034 Nb, 0.067 Ti, 0.0043 N (all in wt %). According to the general assumptions, the high content of alloying elements (e.g., Cr and Mo) in steels results in their strengthening and slowing down the kinetics of softening processes because these elements increase the activation energy of recrystallization. It should also be the case that microalloying elements (e.g., Nb and Ti) bind to carbon or nitrogen in steels and form precipitates (carbides, nitrides or carbonitrides Nb, Ti, etc.). These precipitates are formed at grain boundaries and slip planes, i.e., in places potentially suitable for the nucleation of new nuclei formed by recrystallization. Microalloying elements precipitated in solid solution or precipitates then inhibit recrystallization, increase flow stress and significantly reduce the plastic properties of steels [1,5,37,61,62]. Thus, it can be concluded that a higher strain is required to initiate the dynamic recrystallization of 316LN high alloy steel compared to unalloyed low carbon steel B (with a similar carbon content of 0.16 C), which corresponds to Equations (29) and (23). Similarly, it can also be concluded that in the case of micro-alloyed steels, higher strain is required to initiate dynamic recrystallization than in the case of non-alloyed carbon steels with similar carbon content (0.036 wt % for steel A, 0.16 wt % for steel B), which corresponds to Equations (30), (22) and (23).

### 6.3. Hot Deformation Activation Energy

**Figure 17:**
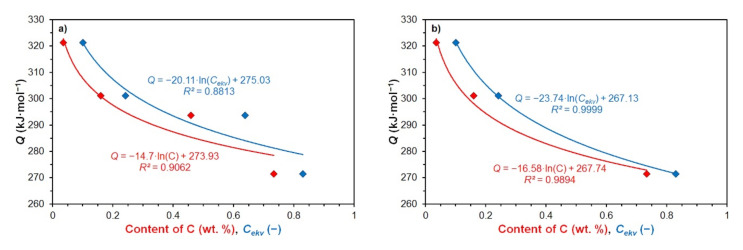
Dependence of hot deformation activation energy *Q* on carbon content and carbon equivalent of investigated steels: (**a**) for all investigated steels; (**b**) for the investigated steels A, B and D.

The determined values of the hot deformation activation energy *Q* decrease with increasing carbon content or with an increasing value of carbon equivalent in the investigated steels, as also documented in Figure 17a:

(31) $$Q=-14.7\cdot \ln\left(C\right)+273.93$$

(32) $$Q=-20.11\cdot \ln\left(C_{ekv}\right)+275.03$$

where *C* is the carbon content (wt %) and *C_ekv_* (−) is the carbon equivalent of the investigated steels. The accuracy of Equations (31) and (32), documented by the relevant coefficients of determination *R*^2^ = 0.9062 for Equation (31), or *R*^2^ = 0.8813 for Equation (32) is sufficient. Suppose that from graphical dependence of the activation energy on the carbon content or on the size of the carbon equivalent is excluded activation energy of medium carbon steel C, because the deformation temperatures of this steel were chosen by a different methodology than in the present work. Thus, the accuracy of simple prediction of the hot deformation activation energy *Q* depending on the carbon content will increase significantly (see Figure 17b):

(33) $$Q=-16.58\cdot \ln\left(C\right)+267.74$$

(34) $$Q=-23.74\cdot \ln\left(C_{ekv}\right)+267.13$$

Excellent accuracy of Equation (33) is documented by the high value of the coefficient of determination *R*^2^ = 0.9894. In the case of Equation (34), the coefficient of determination reaches the value *R*^2^ = 0.9999.

**Figure 18:**
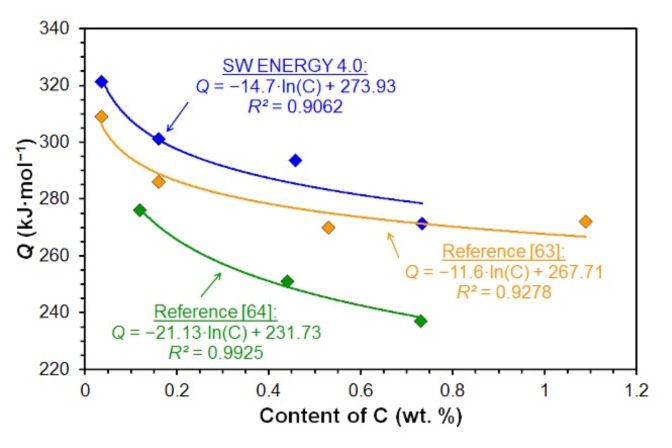
Comparison of values of hot deformation activation energy of non-alloyed carbon steels obtained in the literature research.

The values of hot deformation activation energy *Q* of similar non-alloyed carbon steels can be found in the literature [63,64]. These were subsequently graphically compared with the hot deformation activation energy values determined by analyzing measured data in the ENERGY 4.0 software—see Figure 18. According to Figure 18, the activation energy decrease with increasing carbon content in the investigated steels is in accordance with the work results [61,63,64,65,66]. Mead and Birchenall reported in [67] that the activation energy of iron self-diffusion decreases with increasing carbon content. Carbon increases the self-diffusion coefficient of iron due to the expansion of the lattice that causes it. Thus, the rate of atomic mechanisms controlled by iron self-diffusion may increase with increasing carbon content, such as dislocation climbing, which reduces hot deformation activation energy. Although the exact experimental conditions of the used steels are not clear from the works [63,64], the graph in Figure 18 confirms the logarithmic dependence of the hot deformation activation energy *Q* on the carbon content in non-alloy steels.

Parametric equations expressing the dependence of hot deformation activation energy on the chemical composition of steels can also be found in the literature. In work [64], Elfmark presented a simple equation describing the dependence of activation energy only on the carbon content in non-alloy steels:

(35) Q=288.8−28.6·C

In [62], Medina and Hernandez presented a relationship describing the dependence of hot deformation activation energy on the mass content of carbon, manganese, silicon, molybdenum, titanium, vanadium and niobium:

(36) $$Q=267000-2535.52\cdot C+1010\cdot \mathrm{Mn}+33620.76\cdot \mathrm{Si}+35651.28\cdot \mathrm{Mo}+93680.52\cdot {\mathrm{Ti}}^{0.5919}+31673.46\cdot V+70729.85\cdot {\mathrm{Nb}}^{0.5649}$$

In [68], Colas presented an equation for calculation the value of the hot deformation activation energy of non-alloy steels with a carbon content of 0.03–0.30 wt % C; 0.20–1.70 wt % Mn and max. 0.60 wt % Si:

(37) $$Q=282.7+92.4\cdot C+6.57\cdot \left(\mathrm{Mn}+\mathrm{Si}\right)$$

**Figure 19:**
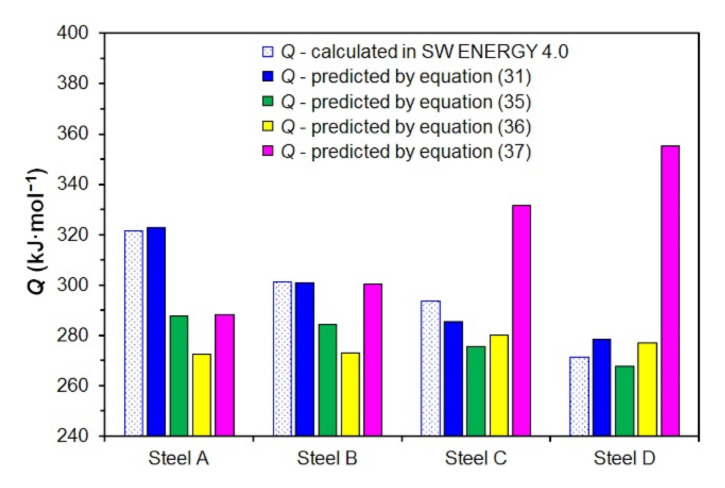
Comparison of values of the hot deformation activation energy *Q* of investigated steels determined by the ENERGY 4.0 software and using Equations (31) and (35)–(37).

A comparison of the activation energy values determined by the analysis of the measured data in the ENERGY 4.0 software and calculated using Equations (31) and (35)–(37) are documented in Figure 19.

Equations (31) and (35) and the results of the analysis of the measured data in the ENERGY 4.0 software reflect the decrease of the hot deformation activation energy *Q* with increasing carbon content in the investigated steels. However, the activation energy determined according to Equation (35) is significantly lower in the case of the investigated low carbon steels—see Figure 19. Medina and Hernandez [62] also included the influence of alloying elements in calculating the hot deformation activation energy *Q*—see Equation (36). This equation was determined by analyzing data measured by torsion tests performed for a wide range of non-alloyed and alloyed steels in strain rates 0.54–5.22 s^−1^ and deformation temperatures 850–1100 °C. The values of activation energy determined according to relation (36) did not differ significantly depending on the carbon content of the non-alloy carbon steels we examined—see Figure 19. According to the comparison chart shown in Figure 19, Equation (36) can only be used for medium and high carbon steel. Colas [68], on the other hand, presented the opposite trend, depending on the carbon content of hot deformation activation energy *Q*—see Equation (37). Unfortunately, work [68] does not mention the thermomechanical forming conditions and chemical composition of the investigated steels, for which the relation (37) was constructed. However, it is clear from the graphical comparison in Figure 19 that relation (37) can only be used to predict the activation energy of non-alloyed carbon steels with a carbon content of about 0.15 wt % C. It follows from the above that parametric Equations (35)–(37) cannot accurately predict the hot deformation activation energy *Q* value for all the steels examined in this paper. For this purpose, the newly introduced relation (31), which expresses the logarithmic dependence of the activation energy on the carbon content in the investigated steels, seems to be more appropriate.

### 6.4. Mathematical Description of Flow Stress Curves of Investigated Steels

The model by authors Cingara and McQueen (14) [27], which was used to derive an equation suitable for predicting critical strain *ε_crDRX_*, can also be used to approximate the flow stress curves of investigated steels. Of course, other models can be used for these purposes, which allow the approximation of the measured flow stress curves in the whole range of strains, such as the model by authors Hensel and Spittel [35], etc.

**Figure 20:**
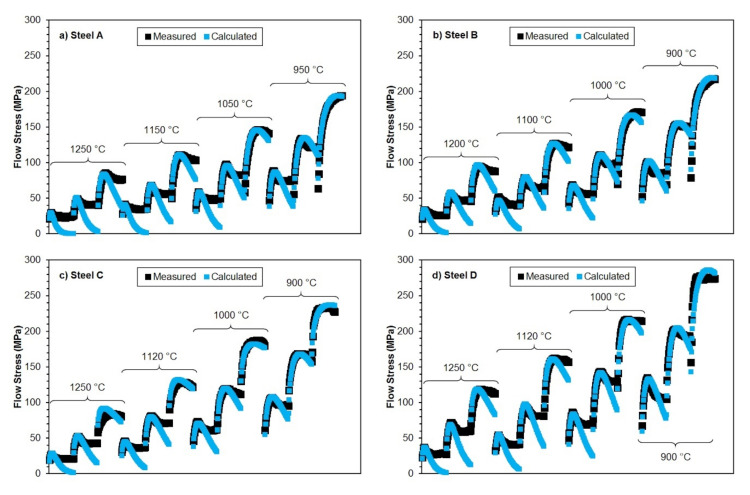
Comparison of measured (black points) and by Equation (14) predicted (blue points) individual flow stress curves of investigated steels: (**a**) steel A; (**b**) steel B; (**c**) steel C; (**d**) steel D.

In this case, however, it is easier to use the Cingara and McQueen model (14) because the values of all relevant material constants are already determined for all investigated steels. Using the predicted values of peak flow stress *σ_p_*, peak strain *ε_p_*, strain hardening exponent *C*, or using the relations (4), (5), (6), (14), (20) and the corresponding material constants (see Table 3 and Table 6), it is, therefore, possible to mathematically describe the individual flow stress curves of investigated steels—see Figure 20. The horizontal axis in Figure 20 is the true strain ranging from 0.02 to 0.94 for each flow stress curve. Individual flow stress curves correspond (for given deformation temperatures) from left to right with strain rates 0.05–1.0–20 s^−1^. If there is no transition to steady state flow (steady state), the model of Cingara and McQueen (14) can be used to approximate flow stress curves with very good accuracy, not only for the range of strains from 0 up to the *ε_p_* (or up to stress *σ_p_*), but also in the area of the prevailing decrease in flow stress—see Figure 20.

**Figure 21:**
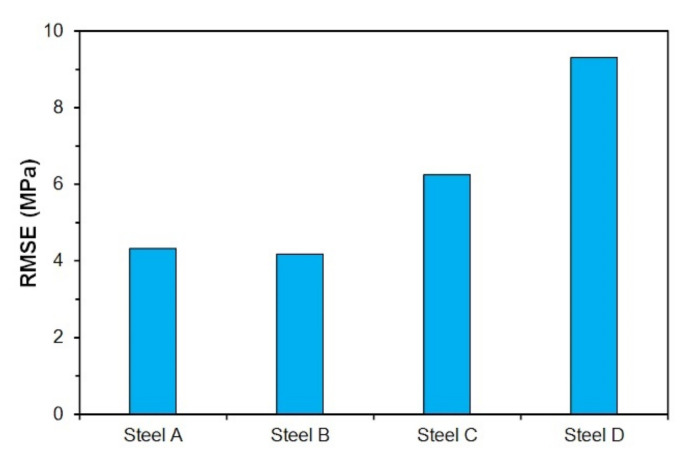
Root mean square error (RMSE) of predicted flow stress curves (by Equation (14)) in range of low strains (from 0 to *ε_p_*) of investigated steels.

The accuracy of predicted flow stress curves can be evaluated using the root mean square error RMSE (MPa) [57,69]:

(38) $$\mathrm{RMSE}=\sqrt{\frac{1}{n}\cdot \sum_{i=1}^{n}{\left[\sigma_{i}-\sigma \left(\varepsilon_{i}\right)\right]}^{2}}$$

where *n* (−) represents the number of points of the flow stress curves of particular steel included in the calculations (together for all combinations of temperature and strain rate). The values of *σ_i_* (MPa) then represent the experimentally obtained values of flow stresses and *σ*(*ε_i_*) (MPa) are the values of flow stresses obtained by prediction through the model (14). The RMSE value then acquires a specific dimension (MPa). Since the model (14) [27] was originally designed to mathematically describe the flow stress curves up to the peak point (up to the strain of *ε_p_*), the RMSE values were determined only for the predicted flow stress curves in the range of strains from 0 do *ε_p_*. The excellent accuracy of the predicted flow stress curves (using Equation (14)) of all examined steels in the range of strains from 0 to *ε_p_* is documented by low RMSE values (see Figure 21), which were up to 10 MPa for all investigated steels. The lowest RMSEs (only about of 4 MPa) are then associated with the steels A and B.

## 7. Conclusions

The stress–strain curves of selected non-alloy carbon steels were investigated using isothermal continuous uniaxial compression tests performed on a Gleeble 3800-GTC simulator.

The obtained stress–strain curves were analyzed in the ENERGY 4.0 software to determine the hot deformation activation energy and other material constants suitable for predicting the peak flow stress *σ_p_* and the peak strain *ε_p_*. The critical strains *ε_crDRX_* required to induce dynamic recrystallization were then determined by analyzing the stress dependence of the work hardening rate on peak stress, using model Cingara and McQueen. In addition, the critical flow stresses *σ_crDRX_* were also determined using this model, which corresponds to the critical strains *ε_crDRX_* of all investigated steels. Based on the achieved results, it was confirmed that for a given size of the Zener–Hollomon parameter, with increasing carbon content (or with increasing value of carbon equivalent) in the investigated steels, their peak flow stress and critical flow stress also increase. In the case of carbon content or carbon equivalent influence, the peak strain and critical strain size made the situation more complicated. However, for all investigated steels, it was confirmed that the difference between the peak strain *ε_p_* and the critical strain *ε_crDRX_* increased with decreasing deformation temperature and increasing strain rate.

Based on the critical strains *ε_crDRX_*, determined according to Equation (14) and subsequently experimentally verified, equations describing with excellent accuracy the simple linear dependence of *ε_crDRX_* on the peak strain *ε_p_* were derived for all investigated steels. In addition, equations comprehensively describing the linear dependence of the critical strain *ε_crDRX_* on the peak strain *ε_p_* and the critical flow stresses *σ_crDRX_* on the peak flow stress *σ_p_* were derived as well.

The determined hot deformation activation energy *Q* decreased with increasing carbon content (or increasing value of carbon equivalent) in the investigated steels, and this dependence was described with good accuracy by a logarithmic equation.

Using the model Cingara and McQueen individual flow stress curves of investigated steels were mathematically described. The excellent accuracy of the predicted flow stress curves of all investigated steels, especially in the range of strains from 0 to *ε_p_*, was documented by the low determined values of root mean square error.

## Figures and Tables

**Table 1:** Chemical composition of investigated steels in wt %.

	Steel A	Steel B	Steel C	Steel D
C	0.036	0.160	0.458	0.733
Mn	0.290	0.370	0.710	0.530
Si	0.036	0.055	0.291	0.220
P	0.009	0.017	0.013	0.009
S	0.010	0.006	0.023	0.013
Cu	0.040	0.040	0.040	0.010
Cr	0.050	0.060	0.270	0.030
Ni	0.030	0.030	0.030	0.010
Mo	0.007	0.011	0.013	0.003
V	0.003	0.005	0.002	0.003
*C_ekv_* (−)	0.101	0.242	0.638	0.830

**Table 2:** Determined temperatures *Ac*_3_, *T_S_* and deformation temperatures *T_d_*.

	Steel A	Steel B	Steel D
*T_S_* (°C)	1522	1496	1379
*Ac*_3_ (°C)	902	862	746
*T_d_* (°C)	1250	1200	1100
	1150	1100	1000
	1050	1000	900
	950	900	800

**Table 3:** Determined material constants intended for prediction of *σ_p_* and *ε_p_* of investigated steels according to Equations (5) and (6).

	Steel A	Steel B	Steel C	Steel D
*Q* (kJ·mol^−1^)	321.37	301.12	293.63	271.42
*n* (−)	5.4113	5.3034	4.7085	4.6809
*α* (MPa^−1^)	0.0083	0.0080	0.0088	0.0075
*A* (s^−1^)	9.59 × 10^12^	2.23 × 10^12^	3.80 × 10^11^	3.81 × 10^11^
*U* (s)	0.00081	0.00240	0.00532	0.00505
*W* (−)	0.198	0.173	0.148	0147

**Table 4:** Accuracy of *σ_p_* values predicted according to Equation (5).

	Steel A	Steel B	Steel C	Steel D
*R* (−)	0.9998	0.9989	0.9993	0.9995
Δ_mean_ (%)	−0.05	−0.04	−0.53	−0.02

**Table 5:** Accuracy of *ε_p_* values predicted according to Equation (6).

	Steel A	Steel B	Steel C	Steel D
*R* (−)	0.9690	0.9352	0.9494	0.8512
Δ_mean_ (%)	−2.53	−4.80	−1.60	−2.39

**Table 6:** The material constants intended for prediction of strain hardening exponent *C* of investigated steels according to Equation (20).

	Steel A	Steel B	Steel C	Steel D
*a* (s^2^)	0.0729	0.0281	−0.0572	−0.0600
*b* (−)	0.9348	0.8252	1.8288	1.4904
*c* (s)	−0.0018	−0.0011	0.0004	0.0020
*d* (−)	−0.0203	−0.0212	−0.0578	−0.0420

**Table 7:** Parameters of additional uniaxial compression tests of steels A, B and D.

	Specimen	*T_d_* (°C)	$\dot{\varepsilon }$	*ε* (−)
Steel A	A1	950	0.05	0.13
	A2	1050	1	0.14
	A3	1150	20	0.18
Steel B	B1	900	0.05	0.16
	B2	1000	1	0.18
	B3	1100	20	0.22
Steel D	D1	900	0.05	0.10
	D2	1000	1	0.12
	D3	1100	20	0.16

## Data Availability

Not applicable.
